# Improved Singing Accuracy in Children With Bilateral Hearing Devices With More Musical Activities and Better Verbal Fluency

**DOI:** 10.1097/AUD.0000000000001786

**Published:** 2026-02-03

**Authors:** Ritva Torppa, Li Xu, Lotta Keitilä, Laura Huhtinen-Hildén, Valerie Looi

**Affiliations:** 1 Cognitive Brain Research Unit (CBRU), Faculty of Medicine, Department of Speech-Language Pathology, University of Helsinki, Helsinki, Finland; 2 Centre of Excellence in Music, Mind, Body and Brain, Faculty of Medicine, University of Helsinki, Helsinki, Finland; 3 Hearing, Speech and Language Sciences, Ohio University, Athens, Ohio, USA; 4 Helsinki Metropolia University of Applied Sciences, Helsinki, Finland; 5 Spiral Therapeutics, San Francisco, California, USA.

**Keywords:** Children, Cochlear implant, Hearing aid, Hearing loss, Informal musical activities, Music intervention, Semantic verbal fluency, Singing pitch accuracy

## Abstract

**Objectives:**

The singing pitch accuracy of children with hearing loss (HL) is typically poor, which may discourage them from singing. Improving this skill is important because it can further impact their cognitive and language development and overall well-being. In children with normal hearing (NH), improving singing pitch accuracy is associated with more musical activities and better language skills. We hypothesized that the singing pitch accuracy of children with HL (1) would be poorer than that of children with NH, (2) would improve more during the music intervention period than during the period without music intervention, (3) would be higher in children who participate in more informal musical activities and (4) would be higher in children who have better semantic verbal fluency performance.

**Design:**

The participants were 18 children with HL (age range: 3 to 6 years 10 months; 7 with bilateral cochlear implants, 7 with bilateral hearing aids, 4 with bimodal devices) and 20 children with NH (age range: 2 years 10 months to 6 years 10 months). Children with HL participated in remote or in-person music intervention for 10 weeks. A crossover study design was used, with waiting periods before and after the intervention. Three rounds of measurements were made for each child with HL, before and after each period (at T1, T2, and T3). Children with NH did not participate in the music intervention, and their singing pitch accuracy was measured at a single time point. Children sang the song “Twinkle Twinkle Little Star.” A measure of mean note deviation assessed the accuracy of each individually sung note, while a measure of mean interval deviation assessed the accuracy of the relative pitch between two consecutively sung notes (e.g., C4 – F4 = 4th), both averaged across the sung sequence. The average of the “mean note” and “mean interval deviation” was used as a dependent variable in a Linear Mixed Model (LMM). For the semantic verbal fluency task, children listed as many animals as they could in one minute, and the number of correct animal words was calculated. Informal music participation was calculated from a parent-completed questionnaire. The mean of the frequency of these activities/number of correct animal words across T1, T2, and T3 was used in LMM.

**Results:**

The singing pitch accuracy of the children with bilateral HL group at baseline (T1) was similar to that of children with NH, and there were no significant effects of intervention versus waiting period on mean note/interval deviation. The LMM showed that (i) the more children with HL participated in informal music activities, the better their singing pitch accuracy (main effect, *p* = 0.009), and (ii) the better they were at the semantic verbal fluency task, the better their singing pitch accuracy (main effect, *p* = 0.014).

**Conclusions:**

Although a 10-week music intervention did not improve singing pitch accuracy in this study of children with HL, these novel results showed that participation in informal music activities, along with language skills, is an important contributing factor to how accurately children with HL can sing in tune.

## INTRODUCTION

Existing research demonstrates that although children with cochlear implants (CIs) and hearing aids (HAs) sing and enjoy singing (Trehub et al. 2009), they often experience difficulty singing in tune (Mao et al. 2013). This may discourage them from singing or limit their opportunities to participate in singing-related musical activities, leading to fewer opportunities for socialization and peer interaction, and potentially lowering their self-esteem or self-confidence. This, in turn, can negatively affect their overall well-being, as well as their cognitive, language, and psychosocial development, and quality of life (Welch et al. 2014; Lo et al. 2022; Papageorgi et al. 2022). With this in mind, the primary aim of the present study was to assess the singing pitch accuracy of children using bilateral hearing devices, aged 3 to <7 years, in comparison to peers with normal hearing (NH), with the secondary objectives of examining the relationship of this singing pitch accuracy to (i) a short-term music intervention, (ii) informal musical activities, and (iii) language skills.

### The Development of Singing Pitch Accuracy in Children With NH

There is a wealth of different theories of the development of vocal singing skills in children. Between the ages of 1 and 5 years, children’s singing is characterized by interaction with their surrounding environment, upbringing, and culture, reflected in different playful, creative, and spontaneous singing behaviors (Welch 2015; Gudmundsdottir 2020). Welch et al. (1997), Welch (2002), and Welch (2015) suggested that pitch-matching develops in children across four phases: (1) singing is in a restricted pitch range with a bias toward lyrics (i.e., words are of more interest than the melody); (2) vocal pitch range expands as do children’s exploration of their ability to control their vocal pitch; (3) melodic shape and relative pitch (i.e., intervals) improve in accuracy, and 4) no significant melodic or pitch errors for relatively simple songs. Davidson (1994) proposed that singing skill development is completed by age 8, however other researchers have suggested that development matures far later, at around 11 to 12 years (Rutkowski 2015; Welch 2015). There are large individual differences in singing skills in the general population (Dalla Bella et al. 2007; Gudmundsdottir 2020), and deficits in pitch perception are not necessarily the only reason for poor-pitched singing (Rutkowski 2015). Singing comprises both physical elements (e.g., breath control, physiological control of the vocal-motor apparatus, lung capacity) and perceptual feedback, the latter requiring the singer to compare between an “internalized voice” used to plan and predict motor instructions for vocal output, and feedback from sensory information that monitors the actual output (Tsang et al. 2011; Pfordresher et al. 2015). Singing also requires both long-term and short-term memory, not just of the song itself, but also for the motor plans required to sing a song. Pfordresher et al. (2015) suggested that many poor-pitch singers may struggle due to an inability to appropriately translate perceptual representations into a motor plan.

In line with the importance of memory, internalized voice, and predictive processes, singing skills can also be improved through practice—both formal and informal (Gudmundsdottir 2020). Yeom et al. (2022) reported that singing informally with family and being surrounded by music early in life are strongly linked to singing pitch accuracy in adulthood. Aside from the physical and physiological considerations related to singing, two other important factors that contribute to singing ability are language skills (as lyrics are an important component of songs/singing), and cognitive ability (Hutchins 2018). For example, a child with better language skills is more easily able to access words efficiently, enabling them to use their cognitive resources to focus on other tasks, such as the pitch patterns of songs or the perceptual feedback loop (Welch 2002).

### Singing Pitch Accuracy in Children With HL

It has been widely reported that children using HAs and/or CIs often have poorer pitch perception skills than children with NH (Looi & Radford 2011; Looi et al. 2012; Limb & Roy 2014; Oxenham 2018), which affects their singing pitch accuracy (Nicastri et al. 2023). It is interesting that Lo et al. (2020) found no significant difference between their children (aged 6 to 9 years) with NH and those with HL (using HAs and/or CIs) in their pitch perception thresholds, with the authors attributing this to the young cohort in the study whose pitch perception skills were unlikely to have been fully matured. Rocca (2012) proposed a schema on the stages of the singing pitch accuracy development of prelingually deafened children after implantation with a unilateral CI, which align with those proposed by Welch (2002) and Welch (2015). Rocca (2012) proposed that at 1 to 5 years after implantation, the focus is on the lyrics rather than the features of the melody, and as language skills develop, the focus shifts more to musical pitch awareness. According to Rocca, only after 6 to 11 years after implantation can a child be expected to imitate melodic contours and pitch intervals and reproduce melodic shapes accurately.

Studies have investigated the singing pitch accuracy of tone-language (where pitch variation can change the meaning of a word) speaking children with HL using acoustic measures examining the deviation of the target notes from the sung notes, fundamental frequency (F0) contour direction of adjacent notes, and the mean interval deviation compared with target notes (Xu et al. 2009; Mao et al. 2013; Yang et al. 2019). Mao et al. (2013) compared singing pitch accuracy of 68 prelingually deafened children (37 children with unilateral CIs and 31 with bilateral HAs, aged 2.1 to 7.2 years) to 37 NH children (aged 3.1 to 6.3 years). Compared with children with NH, children with unilateral CIs and bilateral HAs were less accurate in all pitch-based measures of singing performance, consistent with the results from 7 children with CIs in the study by Xu et al. (2009). For speakers of non-tonal languages, Nakata et al. (2006) compared 12 congenitally deaf Japanese-speaking children with unilateral CIs (aged 4.9 to 10.3 years; mean length of CI use of 3.3 years; 9 used HA in the contralateral ear) to six children with NH (with ages of 6.7 to 9.9 years). Compared with children with NH, the children with CIs produced compressed overall pitch range, random directions of pitch changes (unrelated to the target musical score), and greater pitch interval deviations across the entire song. More recently, Nicastri et al. (2023) studied the singing accuracy in 22 Italian children with CIs (10 with unilateral and 12 with bilateral CIs) aged 7 to 10 years (age at implantation 0.98 to 4.42 years and duration of CI experience 3.83 to 8 years). Compared with 22 peers with NH, children with CIs showed poorer performance in intonation (the ability to sing the right note pitch), melody (the ability to sing a sequence of notes at the right pitch using the right pitch contours and right rhythm), vocal range and memory (the ability to repeat the same sequence of notes in different phrases through the song) when singing a familiar song.

Xu et al. (2022) studied 26 English-speaking children with NH, 13 children with bimodal devices, 31 children with bilateral CIs that were implanted sequentially, and 10 children with bilateral CIs that were implanted simultaneously (all aged between 7 and 11 years). Following the methodologies of Xu et al. (2009) and Mao et al. (2013), they measured the deviation of the target notes from the sung notes, the fundamental frequency (F0) contour direction of adjacent notes, and the mean interval deviation compared with the target notes. The results revealed significantly poorer performance on all pitch-based measures in the three groups of children with CIs in comparison to children with NH. It is interesting that all three groups of children with CIs demonstrated a high degree of individual variability in singing pitch accuracy. Some children with CIs performed relatively well, with their pitch-based measures within the NH range, whereas other children with CIs performed more poorly (Xu et al. 2022).

Previous studies have extensively assessed how device types and other hearing-related factors are associated with the singing pitch accuracy of children with HL. Yang et al. (2019) reported that singing pitch accuracy was better for children with bimodal devices than for children with unilateral CIs in a sample of 10 children, whereas Xu et al. (2022) did not find any differences among children with bimodal devices, those with bilateral CIs that were implanted sequentially or those with bilateral CIs implanted simultaneously (all aged between 7 and 11 years). Mao et al. (2013) also did not find significant differences in the singing accuracy between children with unilateral CIs and those with bilateral HAs in their sample of 68 children with HL (unaided pure-tone average [PTA]: 86.3 ± 15 dB in the better hearing ear). Nicastri et al. (2023) report no significant differences between children using unilateral and bilateral CIs. Xu et al. (2022) reported that although singing accuracy was significantly correlated with unaided PTA (i.e., level of residual hearing) in children in the bimodal group, no demographic or audiological factors were found to predict the vocal singing performance of children with CIs. Nicastri et al. reported that better aided thresholds were correlated with better singing pitch accuracy, with Mao et al. finding age to be a contributing factor. Duration of CI and HA use (hearing age), and age of implantation and/or HA fitting have also been reported to correlate with singing pitch performance (Mao et al. 2013; Nicastri et al. 2023). Hearing age has also been shown to be positively correlated with pitch perception accuracy, an important requirement for singing pitch accuracy (Lo et al. 2020). These results suggest that experience with auditory stimuli (be that from chronological age, hearing age, and/or duration of device use), along with hearing thresholds for children using acoustic hearing, are the most consistent predictive variables associated with singing pitch accuracy for children with HL.

In line with the findings in the NH population, musical activities seem to benefit the development of pitch accuracy in children with HL. Yang et al. (2019) tested the singing pitch accuracy in 10 tonal-language speaking children (aged 7.4 to 12.3 years; 5 bimodal, 5 unilateral CI only) after they had completed 21 months of musical training involving 3 hr of formal choir practice each week, focusing at first on musical scale and once the children could sing the music scale more or less correctly, they started training on popular children’s songs. Each child was also required to practice singing at home every day, approximately 8 hours per week. The singing pitch accuracy results for these children were similar to those of their peers with NH, and significantly better than a comparison group of children with CIs who received no singing training (Yang et al. 2019). These results are in line with those from the study by Welch et al. (2015) suggesting that sustained age-appropriate music education can improve sung pitch range and pitch perception of young children with HL. In Torppa et al.’s (2014b) study, 21 children with unilateral CIs (aged 4 to 13 years) were divided into two groups: (i) children who sang regularly at home (“CI singers,” n = 12) and (ii) those who did not sing regularly (i.e., “CI non-singers,” n = 9). The “CI singers” were significantly more accurate at singing the song “Twinkle, Twinkle Little Star” for rhythm, pitch, and lyrics than the “CI non-singers.” Collectively, these studies suggested that music training and informal musical activities play a role in improving singing pitch accuracy of children with HL. However, there is a lack of studies on the effect of musical interventions on singing pitch accuracy using the measurements typically applied to children with HL, assessing the mean note and mean interval deviation. It is important to note that to the best of our knowledge, no previous study has yet investigated if more informal musical activities are associated with singing pitch accuracy of children with CIs and/or HAs. There is also a lack of studies assessing the role of language skills in singing pitch accuracy.

### The Present Study

In view of the earlier, the aim of the present study is to assess the singing pitch accuracy of children with HL (aged 3 to <7 years) who spoke a non-tonal language (Finnish). We examined the links between informal musical activities and language skills (semantic verbal fluency performance), to their ability to sing in tune. Children with HL participated in remote or in-person music intervention for ~10 weeks (weekly 45 min sessions with home exercises). As the literature has shown that binaural hearing (i.e., bilateral hearing devices) has a significant impact on perceptual and language development for children with HL (Boons et al. 2013), only children with two hearing devices were included in this study.

On the basis of existing research, we hypothesized that the singing pitch accuracy of children with HL:

1. would be poorer than that of children with NH;
2. would improve more during the music intervention period than during the period without music intervention;
3. would be higher in children who participate in more informal musical activities;
4. would be higher in children who have better semantic verbal fluency performance.

## MATERIALS AND METHODS

### Participants

The data collected between September 2019 and May 2022 were impacted by the COVID-19 (Coronavirus Disease 2019) pandemic. The research was conducted in line with the approval from the Helsinki University Hospitals Ethics Committee and a research permit from the City of Helsinki.

The 18 children with HL (HL group; 6 boys and 12 girls, mean age at first measurement 61 months, SD: 15.00, range 36 to 82 months [i.e., 3 years 0 months to 6 years 10 months]) were recruited from a music project organized by the Finnish Association of Cochlear Implant Recipient Children (LapCI). Seven children used bilateral CIs, 7 had bilateral HAs, and 4 had bimodal devices (unilateral CI with contralateral HA) (Table 1). On the basis of the questionnaire, most of the children used their respective devices nearly, if not all of their waking hours, except for one child with bimodal devices who reported using the CI on the left for only a couple of hours per day (but used the right HA for the entire day). It should be noted that for the purpose of this study, “hearing age” (duration of device use) was calculated for HA users from the age when their HA was fitted for the first time, and for the CI users from the age of activation of the CI, which is a widely accepted time frame for calculating hearing age (see for instance Chatterjee et al. 2023). Parental education was categorized using the following scale: 1 Primary education, 2 Secondary education, 3 Undergraduate degree, 4 Bachelor’s degree, 5 Master’s degree, 6 Doctoral degree. The mean parental education level using the mean for both parents in the HL group was 3.56 (SD: 1.16).

**TABLE 1.** Hearing-related background of the HL group and the intervention groups A-B (first intervention, then waiting period) and B-A (first waiting, then intervention period)

	Hearing Age at T1	BiCI	BiHA	Bimodal	Device Model		Age at Diagnosis of Hearing Loss	Age at 1st Device	Aided PTA, Better Ear
	M (SD) (mos)	n	n	n	CI (n)	HA (n)	M (SD) (mos)	M (SD) (mos)	M (SD) (dB HL)
HL group (n = 18)	44.21 (17.84)	7	7	4	Cochlear Nucleus 6 (11)	Phonak (9), Oticon (1), Resound (1)	10.47 (13.75)	13.00 (13.03)	25.00 (3.80)
A-B group (n = 12)	35.17 (17.02)	3	7	2	Cochlear Nucleus 6 (5)	Phonak (7), Oticon (1), Resound (1)	13.75 (15.25)	15.92 (14.68)	25.16 (4.03)
B-A group (n = 6)	58.00 (10.24)	4	0	2	Cochlear Nucleus (6)	Phonak (2)	2.83 (2.40)	6.00 (0.00)	24.80 (3.83)

Hearing age was calculated starting from the age when the first HA was fitted for the child, or if they did not use HA, from the age when their first CI was activated. Age at 1st device = age when children received their first hearing device (HA or CI). Aided PTA, better ear = pure-tone average at 500, 1000, and 2000 Hz with the hearing device (HA or CI) in the better ear, according to the latest available audiograms. The variable age at diagnosis was not normally distributed in the A-B group, and the variable age at first device was not normally distributed in the B-A group. Therefore, the differences between the A-B and B-A groups in these aspects were tested with a Shapiro–Wilk test. Other continuous variables were distributed normally in both groups, and ANOVA was used for these group comparisons.

Bimodal, CI with HA on the contralateral side; CI, cochlear implant; HA, hearing aid; n, number of children; T1, first time point of measurements (the data were collected three times, at T1, T2, and T3); mos, months.

There were also 20 children in the NH group (8 boys, 12 girls; mean age 58.30 months, SD: 12.67, range 34 to 82 months [i.e., 2 years 10 months to 6 years 10 months]), recruited from the kindergartens in Helsinki and through online advertising. The mean parental education level of both parents in the NH group was 4.71 (SD: 0.65), which was higher compared with the HL group (analysis of variance [ANOVA], *F*_1,34_ = 13.91, *p* < 0.001). There was no difference between the HL and the NH groups for age (ANOVA) or sex ratio (chi-square).

### Study Design

**Fig. 1.**
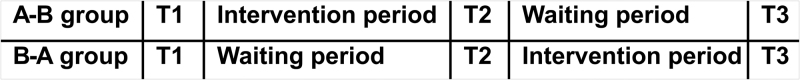
Cross-over study design for children with HL. “A” refers to the intervention, “B” refers to the waiting period. HL indicates hearing loss; T1, T2, and T3, first, second, and third time points of measurements, respectively.

A cross-over study design was used for the children with HL, with some children starting with the intervention while others started in the “waiting period” (no intervention). Children with HL were randomly divided into these two groups (A-B and B-A [see Fig. 1]), where A-B started with the music intervention followed by the waiting period, and vice versa for group B-A. Parents were allowed to change the group if the timing of measurements or intervention was not suitable for them. The details of each group are in Table 1. The A-B and B-A groups differed in duration of device use (i.e., hearing age, ANOVA, *F*_1,17_ = 7.80, *p* = 0.014; children in the B-A group had a higher hearing age), in age when they received their first hearing device (Mann–Whitney *U* = 12.00, *p* = 0.037; children in the A-B group received them at older age than in the B-A group), and in age at diagnosis (Mann–Whitney *U* = 11.50, *p* = 0.027; children in the A-B group were diagnosed at older age than in the B-A group). According to ANOVA, there was no difference in age, parental education, aided PTA at the better ear, or sex ratio (chi-square). The children with NH were tested only once, and they did not participate in the music intervention.

As mentioned, a cross-over study design was used for the HL group. The music intervention was ~10 weeks in duration and is described later. There were small variations in the time between measurements and length of the follow-up (time between T1 and T3) between individual children, however the time between the measurements “before and after” intervention period, and “before and after” waiting period, were similar per child. This was due to illness, school holidays, and the COVID-19 pandemic. The measurements were conducted before and after waiting and intervention periods (i.e., at T1, T2, or T3, respectively), leading to a follow-up time frame of approximately 23 weeks (range 19 to 27 weeks). Parents also completed a questionnaire on the musical activities their child participated in at the start of the study and at T2 and T3 (see later for more details).

### Music Intervention

The music intervention was organized for children with HL only (see Appendix A in Supplemental Digital Content, https://links.lww.com/EANDH/B823). The first (pilot) group (including 3 children in the present study) met face-to-face six times over 6 weeks for 45 min. For the other groups, the intervention comprised 10 weekly group sessions lasting about 45 min, either face-to-face or remote using video conferencing. However, due to the COVID-19 pandemic, the sessions of the spring 2020 group (5 children in the present study) were reduced to eight sessions, with the last session conducted via Zoom video conferencing. The musical activities in the intervention followed the principles given in the article by Torppa and Huotilainen (2019), Huhtinen-Hildén and Pitt (2018), and Huhtinen-Hildén et al. (2024). The activities and reasoning for these are detailed in Appendix A in Supplemental Digital Content, https://links.lww.com/EANDH/B823. The music sessions were always led by a speech therapist and a music pedagogue, along with students from similar fields under the guidance of experienced professionals. The intervention was progressive, with parents/children encouraged to sing and practice at home using the music apps and activities provided to them. Tablet computers with these activities along with music instruments such as claves (percussion instrument, consisting of two sticks, which are played by striking them together) and 5-string kantele (“Finnish zither”; the body of the instrument is made of wood; the steel strings are plucked with fingers) were sent to the family (and returned to the researchers after the intervention period). Obviously, we could not prohibit children/families from singing or conducting their own music activities independently.

### Informal Musical Activities

Parents reported at T1, T2, and T3 the informal musical activities of their children and families using a questionnaire based on those used in Looi et al. (2019) and Torppa et al. (2014a, b); for the musical activity questions, response options, and scales used in the present study, please see Appendix B in Supplemental Digital Content, https://links.lww.com/EANDH/B824. At T1, parents reported the regularity of musical activities before the study began, at T2, the regularity between T1 and T2, and at T3, the regularity between T2 and T3. The regularity of musical activities was assessed using a 9-point Likert scale (0 = not at all/less than monthly to 8 = 8 or more times per day; see Appendix B in Supplemental Digital Content, https://links.lww.com/EANDH/B824). The following questions were used in the “Informal music” variable: Listening to music (audio only); Social music activities (with friends etc.); Listening and watching to musical videos and music programs from TV; Music activities with family; Independent music exploration, Creating/making up songs or music performances for play or fun; Dancing informally; Singing by the child at home (see Appendix B in Supplemental Digital Content, https://links.lww.com/EANDH/B824).

### Procedures for the Measurements of Singing Pitch Accuracy and Verbal Fluency

The vocal pitch accuracy and semantic verbal fluency were measured once for the NH group and at T1, T2, and T3 for the HL group. As mentioned, this study was part of a larger study, and other measures were collected at each of these three time points that are not reported in this paper. Each session was ~ 50 to 60 min (M: 53 min) in duration. For 8 participants with HL, face-to-face measurements were conducted at T1 and T2 in a quiet room at the University of Helsinki. For these participants at T3, and for all other participants (the other 10 HL children, all NH children), measurements were conducted remotely via videoconferencing (Zoom); the Zoom settings and all hearing device settings were kept similar across the time points due to COVID-19 restrictions. In the remote sessions, parents were not allowed to help the children during the actual tasks, but were required to monitor that the sound quality was clear for all measurements and report if they heard any disruptions to the sound quality, verify audibility of task instructions given by the tester (lipreading allowed), and could repeat the task instructions to their child if required. Parents were asked to confirm that their child’s hearing devices were adjusted to normal everyday settings and that their computers, loudspeakers, and Zoom settings were similar to the previous measurements.

In face-to-face measurements, parents were not present in the session itself. Logopedics students conducted the session measurements using Lenovo ThinkPad T14 computers, high-quality sound cards (Focusrite Scarlett Solo 3rd Gen, 2-Channel USB2), and loudspeakers (Fostex 6301NB Active Monitor placed in front of the child at 70 cm from their ears). In the remote measurements, similar computers were used by the tester (students of logopedics or the first and third authors of the present study). However, the computers and loudspeakers used by children with HL and NH in the remote measurements varied according to what the family had at home due to the restrictions and “shut down” resulting from the COVID-19 pandemic.

The Ling six sound test (auditory only) was performed by the experimenter (in face-to-face and remote sessions) before conducting the measurements to ensure that the participants could hear and discriminate speech sounds and task instructions (Ling 1976). In the remote measurements, parents were instructed to set the speaker sound level so that it sounded like speech from a distance of 1 meter, from the place where their child was seated for the session. Then the examiner said the Ling sounds /a/,/i/,/u/,/m/,/s/,/sh/, and the child repeated and/or pointed to pictures of animals that represented the sounds (pictures were familiarized for the children before the actual measurement if they did not know them beforehand). All children participating in the present study could accurately repeat or point to the pictures at all time points. The singing and verbal fluency performance of each child was recorded in face-to-face sessions with a high-quality audio recorder (Zoom H6 Handy Recorder, microphone at 50 cm distance at 45° angle from the child, left or right) and in remote measurements with this (recording microphone placed near the speaker system) and using Zoom video conferencing (computer’s microphone, placed in front of, at ~50 cm distance from the child and the tester), to allow for subsequent data analyses.

### Singing Pitch Accuracy Analyses

The children were instructed to sing the song “Twinkle Twinkle Little Star,” a highly familiar song in Finland, also used in the study by Torppa et al. (2014b). The parents and children were informed of this requirement before the first session, to sing/practise this song with their child (we confirmed with parents that they and the child knew the song before inclusion in the study), and there was no imitation (i.e., no initial presentation of the song) nor any accompaniment. The collected singing samples varied in the total number of notes, as not all of the children sang the entire song. The minimum number of notes across the samples was 6; all notes sung by the child were included in the analyses. Acoustic analysis of the singing pitch accuracy followed the methodology in Xu et al. (2022). First, individual notes were extracted from the audio file, from which the fundamental frequency (F0) of a stable portion of each vowel in the sung note was derived using a custom MATLAB program with an autocorrelation algorithm. The average F0 for each note served as the pitch estimate. Next, we converted the pitch of each note (mean F0 in Hz) to semitones relative to middle C using the formula: semitone = 12 × log_2_ (F0/261.6). Subsequently, for each song, we normalized the F0 values (in semitones) to the mean across all notes in that song. Essentially, we subtracted the mean semitone of the song from each note’s semitone. This process ensured valid comparisons across songs sung at varying pitch levels. We also applied the same normalization process to the target notes in the musical scores.

To evaluate the singing pitch in the melodies sung by each participant, we calculated two pitch-based metrics using the normalized F0s in semitones:

1. Mean note deviation: This metric assessed the accuracy of each note by computing the absolute difference in semitones between the sung note and its corresponding note in the target musical score. The mean deviation across all notes in the song indicated overall pitch accuracy, with a value close to zero suggesting precise pitch matching.
2. Mean interval deviation: This metric evaluates pitch changes between adjacent sung notes compared with those in the target song. By calculating the difference in pitch intervals (in semitones) and averaging across the sequence, we assessed how closely the pitch changes in the sung version matched those in the target music. A mean interval deviation close to zero indicated similar pitch changes between the sung and target versions.

To simplify the statistical analyses, metrics 1 and 2 were combined into one metric by calculating their mean. This metric is referred to as the ‘mean note/interval deviation’ from this point forward.

### Semantic Verbal Fluency

In the semantic verbal fluency task, task instructions asked the child to list names of animals from memory for 60 sec (Tallberg et al. 2011; Pekkala 2012). This task, widely used both clinically and for research purposes in children younger than 6 years old, was chosen because it reflects the efficiency of verbal recall, access to vocabulary (e.g., lexical access), and production of words (Isacoff & Stromswold 2014; Ruffini et al. 2023), necessary during singing songs with lyrics. The instruction given for the child was (translated from Finnish to English): “Tell me all the animals you know. They can be any kind of animals, but don’t say the same animal twice. And try to come up with as many animals as possible. What kind of animals do you know?” The number of correct words (the words belonging the given semantic category “animals”) was calculated to assess the efficiency of semantic verbal fluency performance. If the children produced two words whose content could mean different things to them (in Finnish, leopardi—pantteri, English translation: leopard—panther), a point was given for both outputs. If the child produced synonyms, words that were clearly similar in terms of their semantic content (e.g., pig—pig) or morphological variants of the same word (shirt—shirts), it was interpreted as perseveration, and only the second word was given a point (Troyer 2000).

### Statistical Analyses

Hypothesis 1 was tested with an analysis of covariance (because the NH group was tested only once), with the mean note/interval deviation as dependent variables, group (NH versus HL group) as a fixed factor, and age was controlled due to the large variation of participant ages in this study.

The other three hypotheses were tested in the HL group using Linear Mixed Models (LMM), as this model accounted for missing data. Because the analysis of regression and multi-factor models requires a sample size that is large relative to the number of terms on the model, it was not feasible to use a single model that concurrently included all of the potential predictors of singing pitch accuracy and their interactions. Therefore, a set of smaller models was selected to test specific hypotheses. Only two-way interactions were included in the models due to the restricted amount of data. For all analyses, nonsignificant interactions were dropped from the final models (Torppa et al. 2014a,b). A compound symmetry covariance structure was used. Hearing age was controlled (added as a covariate into the model analyses).

For hypothesis 2, a difference score was calculated by calculating the difference in the mean note/interval deviations before and after intervention, and before and after the waiting periods. These difference scores were then used as a dependent variable, “intervention versus waiting period” as a fixed factor, and hearing age as a covariate in the LMM model.

Hypothesis 3 was tested using mean note/interval deviation as the dependent variable, time (T1, T2, and T3) as a fixed factor, and to simplify the model and analysis, the mean of informal music at T1, T2, and T3 and hearing age as covariates in the LMM model.

Hypothesis 4 was tested using mean note/interval deviation as a dependent variable, time (T1, T2, and T3) as a fixed factor, and the mean of the number of correct words for animals at T1, T2, and T3 and hearing age as a covariate. Regarding the means of informal music activities and the number of correct words, if data for an individual child were missing at any time point, the mean was calculated across the time points where data were available. Because duration of follow-up and with it, the time between the measurements T1 and T2, and T2 and T3, varied between children with HL, we also analyzed if the time between T1 and T3 (in days) correlated with the mean note/interval deviation difference scores between T1 and T3, and the difference scores before and after intervention, and before and after waiting periods across A-B and B-A groups. The correlations were not significant (*p* ≥ 0.355), suggesting that the variation in the time between T1 and T3 or intervention and waiting periods did not play a role in the development of singing pitch accuracy, and these were not included in the LMM.

The critical level for significance was set at 0.05. Bonferroni correction was used for post hoc analyses. All statistical analyses were performed with IBM SPSS 28.0 software.

## RESULTS

**Fig. 2.**
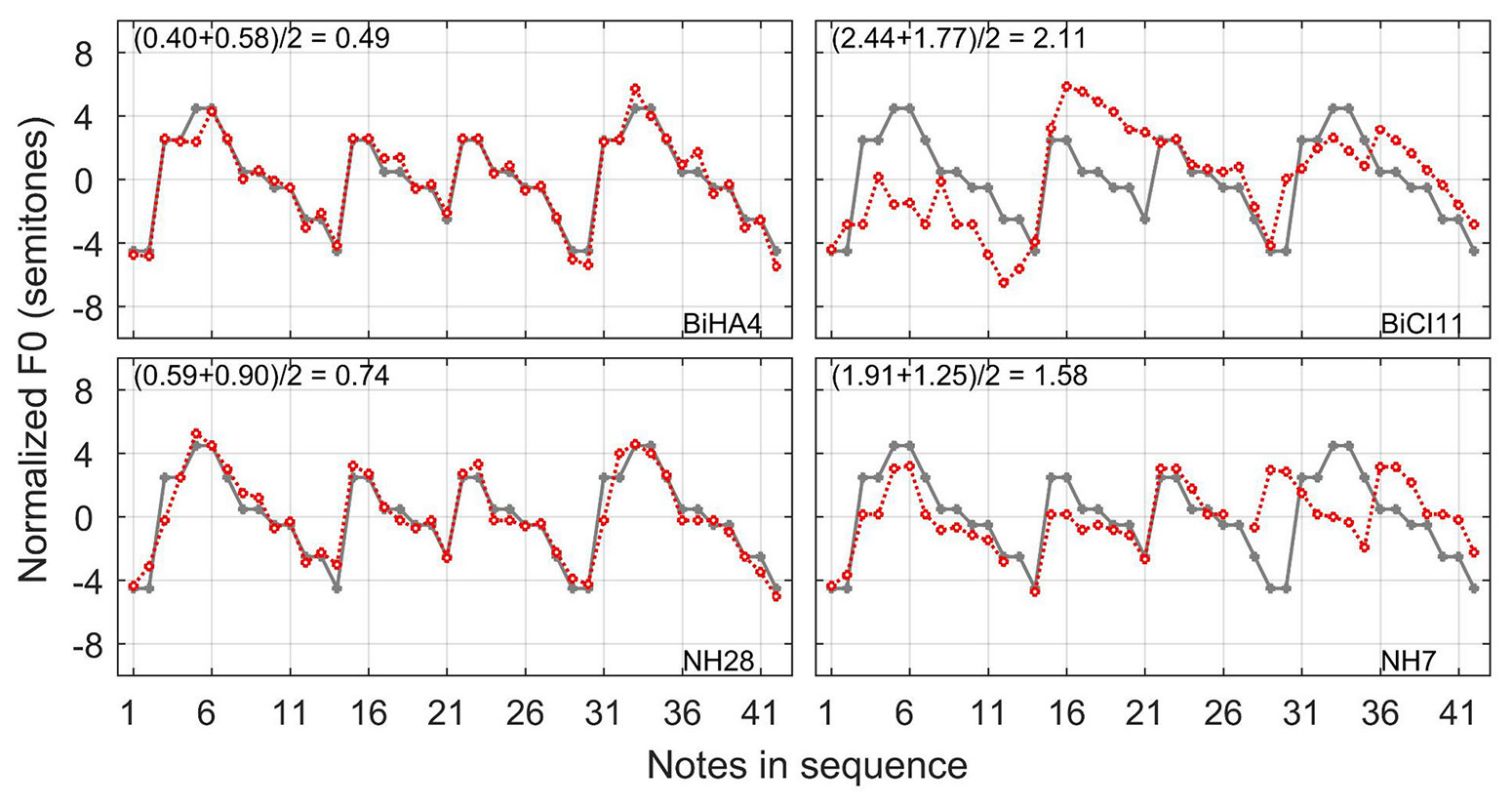
Pitch contours of two children from the HL group (upper row) and two children from the NH group (lower row). The two children on the left were selected from the relatively good performers of each group. The two children on the right were selected from the relatively poor performers of each group. In each panel, the gray solid line represents the target contour and the red dotted line represents the sung contour. The numbers in the upper left corner represent (mean note deviation + mean interval deviation)/2 = mean note/interval deviation in semitones. HL indicates hearing loss; NH, normal hearing.

The singing pitch accuracy of children with NH and HL varied considerably. Figure 2 illustrates examples of pitch contours of four children, two from each group, respectively. For each group, one relatively good performer and one relatively poor performer were selected. The good performers’ pitch contours closely match the target contour (Fig. 2, left panels), indicating nearly perfect singing performance based on pitch accuracy. The poor performers’ contours deviate from the target contour by a number of semitones (Fig. 2, right panels; mean note deviation for the child with HL [BiCI11] was 2.44, and that for the child with NH [N7] was 1.91 semitones), indicating imprecise pitch production.

### Hypotheses 1 (HL Versus NH Group) and 2 (Intervention Versus Waiting Period Comparisons)

Table 2 shows the descriptive statistics for the mean note and interval deviation for the HL and NH groups. For the HL group, there was no clear improvement over time or between measurements. The lack of improvement over time was confirmed with LMM, where the dependent variable was mean/note interval deviation at T1, T2, and T3, and time was a fixed factor (main effect of time, *p* = 0.182). Also, the difference scores before and after the intervention period (M: 0.13, SD: 0.44) and before and after the waiting period (M: 0.11, SD: 0.59) were similar.

**TABLE 2.** Descriptive statistics for the vocal singing pitch accuracy variables at T1, T2, and T3 for the song Twinkle Twinkle Little Star, given separately for the subgroups children with BiCIs, children with BiHAs, and children with bimodal devices (one CI and one HA), as well as for all children with HL (group) and NH (group)

	BiCIs, Mean (SD) (n*)	BiHAs, Mean (SD) (n†)	Bimodal, Mean (SD) (n‡)	HL Group Mean (SD) (N§)	NH Group, Mean (SD) (N = 20)
Mean note deviation in semitones					
T1	1.76 (0.69)	1.09 (0.75)	1.03 (0.08)	1.30 (0.70)	1.41 (0.48)
T2	1.97 (0.68)	1.58 (0.86)	1.13 (0.23)	1.69 (0.75)	
T3	1.58 (0.51)	1.74 (0.69)	1.33 (0.49)	1.57 (0.54)	
Mean interval deviation in semitones					
T1	1.45 (0.51)	1.17 (0.89)	1.23 (0.17)	1.28 (0.66)	1.17 (0.49)
T2	1.51 (0.54)	1.52 (1.15)	0.79 (0.07)	1.43 (0.84)	
T3	1.44 (0.37)	1.30 (0.30)	1.06 (0.30)	1.32 (0.35)	
Mean note/interval deviation in semitones					
T1	1.60 (0.59)	1.13 (0.80)	1.13 (0.12)	1.29 (0.66)	1.29 (0.45)
T2	1.74 (0.59)	1.55 (0.97)	0.96 (0.15)	1.56 (0.76)	
T3	1.51 (0.40)	1.52 (0.47)	1.20 (0.40)	1.44 (0.41)	

* Number (n) of children with BiCIs, at T1 = 5, at T2 = 7, at T3 = 7.

† n children with BiHA, at T1 = 7, at T2 = 7, at T3 = 4.

‡ n children with 1HA and 1CI (bimodal), at T1 = 3, at T2= 2, at T3 = 3.

§ N (number of children) in the HL group at T1 = 15, at T2 = 16, and at T3 = 14.

T1, T2, T3 = the first, second, and third time points of measurements.

Contrary to hypotheses 1 and 2, an analysis of covariance showed no differences between HL and NH groups, and the LMM did not show significant effects of intervention versus waiting period for mean note/interval deviation.

Although not specific to these two hypotheses, one observation worth pointing out is the trend noted in singing accuracy scores between children who used bilateral CIs compared with those who used acoustic hearing (either bilateral HAs or bimodal stimulation) (Table 2). Although the “n” for each subgroup was too small to perform statistical analyses, a clear trend can be seen that the mean note/interval deviation was larger (i.e., worse) for the children using bilateral CIs compared with the other groups, particularly at T1 and T2.

### Hypothesis 3 (Informal Musical Activities)

Table 3 shows descriptive statistics for the regularity of informal musical activities of the HL group separately for each question at T1, T2, and T3 and for their mean across these three time points. The most regular activities were making music performances for play or fun (~ 4 to 6 times per week at T1, T2, and T3) and singing by children themselves (~ 1 to 3 times per day at T1, T2, and T3) (see questions 6 and 8 at Table 3). Children listened to music ~ 3 to 4 times per week, danced informally, and watched musical videos ~ nearly 2 to 3 times per week. They also participated in social music activities with friends or siblings and made independent music explorations with self-made music instruments weekly (Table 3).

**TABLE 3.** Individual item means and standard deviations for informal music activities included in statistical analyses at each time point of measurement (T1, T2, and T3) for the children with hearing loss (HL group)

	1. Listening to Music (Audio Only)			2. Social Music Activities			3. Musical Videos			4. Family Music Activities			5. Independent Music Exploration		
	T1	T2	T3	T1	T2	T3	T1	T2	T3	T1	T2	T3	T1	T2	T3
n	16	15	16	15	15	16	16	16	16	16	16	14	16	15	15
M	4.62	4.73	4.53	3.60	3.40	2.53	3.69	3.81	4.25	1.81	2.19	1.64	2.94	3.20	3.00
SD	1.75	1.34	1.61	1.50	1.72	1.95	1.40	1.56	1.24	1.83	1.56	1.55	2.08	2.37	1.56

	6. Music Performances			7. Dancing Informally			8. Singing by the Child						Mean of Musical Activity Questions		
	T1	T2	T3	T1	T2	T3	T1	T2	T3				across T1, T2, and T3 = “Informal Music”		
n	16	15	14	16	15	16	17	16	16				17		
M	4.94	5.33	5.07	4.19	3.87	3.88	6.00	5.90	5.75				3.95		
SD	1.53	1.40	1.73	1.80	2.03	2.10	1.54	1.37	1.39				0.92		

A 9-point Likert scale was utilized (0 = less than once in a month; 1 = once in a month; 2 = 2–3 times per month; 3 = weekly; 4 = 2–3 times per week; 5 = 4–6 times per week; 6 = 1–3 times per day; 7 = 4–7 times per day; 8 = more than 8 times per day. For detailed information on the questions, response options, and scales, see Appendix B in Supplemental Digital Content, https://links.lww.com/EANDH/B824. The variable “Informal music” was used for testing hypothesis 3.

**Fig. 3.**
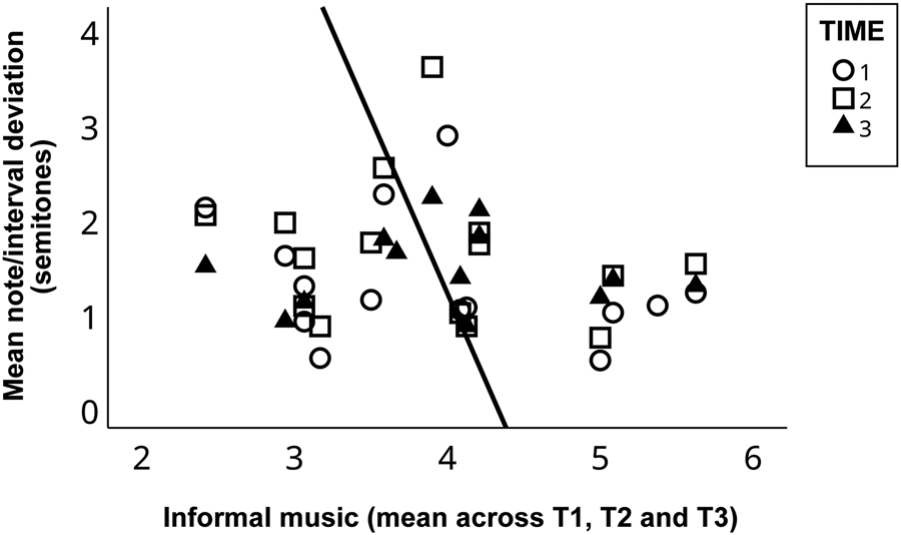
Illustration of the results for hypothesis 3 showing the direction of the connection of mean/note interval deviation (in semitones) at T1 (circles), T2 (squares), and T3 (triangles) with the mean of informal musical activities across T1, T2, and T3 (“Informal music,” for the scale, please see earlier and Table 3). Please note that (A) the smaller the value for mean note/interval deviation, the more accurate the singing pitch accuracy of the child; (B) the figure does not consider hearing age controlled in the LMM model. LMM indicates Linear Mixed Model.

When testing hypothesis 3, there was a significant main effect of “informal music” (*F*_1,13_ = 5.93, *p* = 0.009, *B* = −1.310) for mean note/interval deviation at T1, T2, and T3. The connection (*B*) was negative, suggesting that the intervals deviated less from the original ones (reflecting better singing pitch accuracy) with more informal music (Fig. 3). Also, the main effect of hearing age was significant, *p* = 0.008, with the connection being negative, suggesting better (smaller) mean note/interval deviations with greater hearing age. The significant interaction of hearing age and informal music (*p* = 0.015) indicated that the connection was stronger in children with younger hearing age.

### Hypothesis 4 (Semantic Verbal Fluency)

Appendix C in Supplemental Digital Content, https://links.lww.com/EANDH/B825, shows descriptive statistics for semantic verbal fluency performance at T1, T2, and T3 and their mean across these three time points. The mean of the number of correct words (animals) children produced during 60 sec was 8.46, and there was high variability in the performance of children with HL at all three time points (see Appendix C in Supplemental Digital Content, https://links.lww.com/EANDH/B825).

**Fig. 4.**
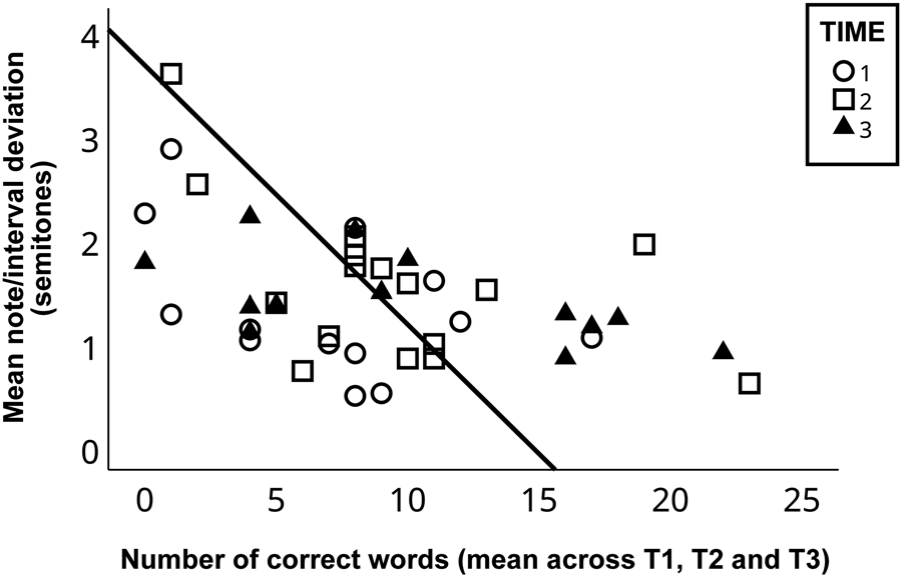
Illustration of the direction of the connection of mean/note interval deviation (in semitones) at T1 (circles), T2 (squares), and T3 (triangles) with verbal fluency performance (the mean of the number of correct words the child listed across T1, T2, and T3). Please note that (A) the smaller the value for mean note/interval deviation, the more accurate the singing pitch accuracy of the child; (B) the figure does not consider hearing age in the LMM model. LMM indicates Linear Mixed Model.

There was a significant main effect of the number of correct words for mean note/interval deviation at T1, T2, and T3 (*F*_1,12_ = 8.27, *p* = 0.014, *B* = −0.204); connection (*B*) was negative suggesting that the intervals deviated less from the original ones (reflecting better singing pitch accuracy) with better semantic verbal fluency (see Fig. 4, for the illustration of the direction of the connection). The main effect of hearing age was significant, *p* = 0.032, with a negative sign, suggesting better (smaller) mean note/interval deviations with a greater hearing age. The significant interaction of hearing age and semantic verbal fluency (*p* = 0.036) indicated that the connection was stronger in children with younger hearing age.

## DISCUSSION

The present study assessed whether singing pitch accuracy of children aged 3 to <7 years with bilateral HL using bilateral hearing devices would differ from their peers with NH (hypothesis 1), and if children with HL would improve in this skill when participating in music intervention, as compared with periods without music intervention (hypothesis 2). We also assessed if the ability of children with HL to sing in tune would improve with more informal musical activities (hypothesis 3) or better semantic verbal fluency performance (hypothesis 4). Hypotheses 1 and 2 were not supported as the singing pitch accuracy of children with HL did not differ from children with NH and we did not find more improvement during the music intervention period than during waiting periods. However, the results supported hypotheses 3 and 4. The singing pitch accuracy of children with HL improved with more informal musical activities and better semantic verbal fluency performance.

Unexpectedly, and contrary to previous reports (Mao et al. 2013; Nicastri et al. 2023), the singing pitch accuracy of children with HL in this study was not significantly different from that children with NH. Similarly, contrary to existing findings for children with NH, music intervention did not improve the singing pitch accuracy of children with HL. There may be a few reasons for this. First, the children with HL’s performance was similar to the NH group at T1, and due to this ceiling effect, we would not expect any improvement in the HL group during our follow-up or 10-week music intervention because it is not reasonable to expect that the children with HL would be significantly better than their NH peers at this task. One possible reason for the children with HL’s initial good scores is that our cohort of children participated in informal musical activities before the study started. This is similar to the study by Yang et al. (2019), where the children had had 21 months of choir training before the first study measurements, and their initial results showed similar performance levels to the NH group. This also aligns with the results of Hypothesis 3 where better singing pitch accuracy was found for children who participated in more informal activities.

Another potential reason for the null findings for hypotheses 1 and 2 could be the young age of the participants. Previous research (Welch 2015; Lo et al. 2020; Papageorgi et al. 2022) suggests that young children are less accurate in their pitch production and perception, as their maturation continues at an older age (for pitch production, at the age of 8 to 12 years, Rutkowski 2015; Welch 2015). For example, the children in Lo et al.’s (2020) paper were aged 6 to 9 (slightly older than the children in this study), and the authors of that study reported that even their NH children’s pitch perception skills were unlikely to have fully matured. The null finding for hypothesis 2 is also consistent with the findings of Looi and Radford (2011) as well as Lo et al. 2020, where 12 weeks of music training did not improve pitch perception skills in their children aged 6 to 9. Other potential reasons for the null finding for both hypotheses 1 and 2 may be related to the study design, and that our HL group combined children using electric-only hearing, acoustic-only hearing, and bimodal stimulation. Many studies have reported that the use of an HA often results in better pitch perception (which then may translate to better pitch production) in both children and adults (Looi & Radford 2011; Looi et al. 2012; Nicastri et al. 2023; Bleckly et al. 2024). In the CI speech processing strategies used in those studies, fine-structure temporal information is discarded with only amplitude-envelope information being retained; this fine-structure information has been shown to be important for pitch perception (Moore & Carlyon 2005). HAs, which use acoustic stimulation as opposed to the electrical stimulation of CIs, retain (at least partially) this fine-structure information, which helps with the perception of pitch (Moore & Carlyon 2005; Limb & Roy 2014). Combining all of the device configurations into one group may have reduced the sensitivity of finding whether training could have shown benefit for children using one device configuration (e.g., electric only hearing) over another (e.g., acoustic only hearing). One other possible reason is that one of the inclusion criteria was that the child knows, and is able to sing “Twinkle Twinkle Little Star” which may have unintentionally biased recruitment to children with better singing or pitch perception skills.

As mentioned, hypothesis 3 was supported. The most regular activities of the children with HL were making music performances for play or fun (approximately 4 to 6 times per week) and singing by children themselves (approximately 1 to 3 times per day). They also participated in social music activities with friends or siblings, made independent music explorations with self-made music instruments weekly, listened to music, and watched musical videos. Thus, these activities give lots of opportunities to hear, imitate, and sing in the correct pitch. These musical activities, which intertwine the sense of agency and motivation, social context, positive emotion, and interaction, are expected to support singing skills (Huhtinen-Hildén & Pitt 2018; Gudmundsdottir 2020; Huhtinen-Hildén et al. 2024) as well as vocabulary, prosocial skills, and attentional regulation (Williams et al. 2015). It is possible that in childhood, there is a critical period that allows individuals with NH to achieve high precision in their use of singing voice for correctly reproducing vocal music of the culture (Gudmundsdottir 2020). The present results, showing both similar singing pitch accuracy between children with HL and NH and improvement with more informal musical activities among participants younger than 7 years of age, raise the question of whether there could also be a similar critical period for children with HL. Our results are also in line with those from a large-scale “Sounds of Intent” project (https://soundsofintent.org/en/research-and-development/outputs), showing that appropriate musical experience can support musical development across the lifespan for a range of different conditions and disabilities.

We found support for hypothesis 4 with better singing pitch accuracy of children with HL being linked to better semantic verbal fluency performance, which reflects the effectiveness of lexical access and verbal recall (Pekkala 2012). This is in line with the previous findings of Hutchins (2018), showing that performance in rapid naming and phonological awareness tests correlates with singing pitch accuracy in children with NH, and with theories on the development of singing pitch accuracy in children with NH (Welch et al. 1997; Welch 2015). The former and present findings indicate that if a child with NH or HL is more easily able to access words efficiently, they are able to use their cognitive resources to focus on other tasks, such as the pitch patterns of songs or the perceptual feedback loop. The link between the semantic verbal fluency and singing pitch accuracy could also be bidirectional. For example, improving singing pitch accuracy may improve semantic verbal fluency, and/or improving semantic verbal fluency may improve singing pitch. Further still, even though the present study did not investigate pitch perception, improving singing pitch accuracy may help to improve pitch perception, which in turn could lead to better language skills, verbal recall, and semantic verbal fluency performance. Research studies at the level of the brain have shown that pitch perception is not processed only in temporal auditory areas, but it is also related to the efficiency of the dorsal and ventral pathways, which appear responsible for automatic, category-based sound analysis and conscious access to perceptual information. Results of Loui et al. (2009) have shown that these pathways are weaker in adults with amusia and poor singing pitch accuracy, while Halwani et al. (2011) showed that these pathways are more effective with more singing and better pitch perception. Torppa et al. (2014b) found that children with CIs who sang more had improved P3a responses, which are related to effective connections between frontal and temporal auditory areas. Again, the direction of these relationships, and the degree of any bi-directionality, is unknown. Regardless, it does raise the possibility that singing more could lead to improved connections in the brain, which may subsequently lead to better pitch perception.

It should also be considered that if a child can better perceive pitch, this has been shown to translate to language acquisition in general (Cole 2015). For children with CIs, better pitch perception is connected with better prosodic stress perception (Torppa et al. 2014a), which in turn is connected to better word finding skills, higher verbal IQ and better phonological awareness (Torppa et al. 2020). This proposal of bi-directionality between pitch or singing and language is also supported by the findings in older children with NH, showing that a pitch-based singing training program for preschoolers that focused on singing intonation improved phonological awareness (Patscheke et al. 2019). According to the results from the Sing Up Program, singing can also improve the overall well-being of children (Papageorgi et al. 2022). One key takeaway message from all of this is that the research evidence does indicate that informal musical activities can improve a wide range of abilities beyond just music skills, and hence parents and clinicians should try to integrate more informal music participation into a child’s everyday routine. Another option is to have the family (child with parent(s)/caregivers) participate in professionally facilitated group music activities, which can help the families to create a supportive environment where a child feels able/comfortable and motivated to sing (regardless of their singing ability).

## LIMITATIONS AND FUTURE DIRECTIONS

The findings of this study should be interpreted in consideration of some limitations. First, our sample size was small, and as a result, the HL group combined different device configurations. This prevented us from analyzing for differences between electric and acoustic hearing, which has been shown to often be a significant contributor to pitch perception. Future studies with larger sample sizes could evaluate for these differences while accounting for any potential contribution of music activity participation and/or semantic verbal fluency skills.

The finding that the children with HL performed similarly to children with NH could be a consequence of other factors than those discussed earlier. Perhaps our results and those from a recent study by Lo et al. (2020) showing similar pitch perception accuracy between children with HL and NH are a reflection of current/modern early intervention practices and a greater awareness of the needs of children with HL. These include early diagnosis, early bilateral hearing device fitting and activation, new/upgraded devices, better support for parents/caregivers, evidence-based pedagogical methodologies (such as auditory verbal therapy) for early intervention practices, improved training and upskilling for professionals, to name only a few (Hammer et al. 2024). Research has not yet determined whether there is a critical period for the development of pitch perception in children with NH, let alone children with an HL, and this would be a valuable study to conduct.

The results for the music intervention could also have been affected by the variation in the settings for music intervention, with some conducted remotely and others face-to-face, or the intensity (one 45-min session per week) or length of the intervention (10 weeks only). Learning tends to be better with a greater number of sessions over longer time periods (Moore & Amitay 2007; Kraus et al. 2009). Moreover, there was missing data at each time point of measurements for the children with HL, which may affect the findings. The missing data could not be avoided because the participating children were young (<7 years), and it was not possible to force them to sing. However, having three time points of measurements increased the number of singing samples, which was evidently beneficial for finding the main effects we observed.

One potential future study is to investigate the role of informal music activities on singing pitch accuracy and/or verbal fluency performance. A future study could implement a structured but flexible program of informal music participation. For example, based on the levels of informal music activity participation before starting in the study, parents could be asked to double this participation level for 6 months, and children reevaluated after 6 months. The parents/children could choose how this was implemented into their daily lives (e.g., more music played at home, more singing or musical play activities), but are required to keep a music diary for the duration of the activities utilized.

Last, it is important to keep in mind that the relationships between variables found in this study are correlational and bidirectional, and not causal. As with any correlation, the potential of other confounds or contributory variables must also be considered.

## CONCLUSIONS

The results from the present study suggest that singing pitch accuracy in children with bilateral HL and bilateral devices is better with more informal musical activities and better semantic verbal fluency performance. Poor singing pitch accuracy can discourage children from singing, limit their opportunities to participate in singing-based musical activities, and result in reduced opportunities for socialization or interacting with peers. This can further lead to poorer self-esteem or self-confidence, psychosocial well-being, or quality of life, and poorer language skills, the latter supported by this study’s findings, which showed that singing pitch accuracy was associated with verbal fluency performance. It is hoped that the results of this study will encourage parents/caregivers/families of children with HL to include more musical activities into their everyday life, as well as provide the basis for future research to explore the relationship between informal music activities, singing pitch accuracy, and verbal fluency, and the impact of electric and/or acoustic hearing on these variables in larger studies.

## ACKNOWLEDGMENTS

The authors thank the laboratory engineers Jaakko Kauramäki and Tommi Makkonen for their assistance in matters related to the data collection, the students who collected data, the Head of Department at Hospital District of Helsinki and Uusimaa (HUS), Associate Professor Antti Aarnisalo for his help with the approval from the HUS Ethics Committee, Associate Professor Seija Pekkala for her help with the semantic verbal fluency task and the Finnish Association of Cochlear Implant Recipient Children (LapCI) for organizing the music intervention. Ohio University student, Victoria Costa, provided technical assistance in acoustic analysis. Most of all, the authors want to thank the participating children and their families.

## Supplementary Material
