# Supplementary material for: Improved Singing Accuracy in Children With Bilateral Hearing Devices With More Musical Activities and Better Verbal Fluency
Source: Ear Hear. 2026 Feb 3;47(4):925–35. doi: 10.1097/AUD.0000000000001786 (PMC13252975; doi:10.1097/AUD.0000000000001786)
Supplement: Supplementary file 1 [file aud-47-0925-s001.pdf]

Appendix A: Description of the intervention

Before beginning of the music sessions, parents were interviewed by the students of logopedics (speech and language pathology in guidance of professional speech and language therapist) and background information especially concerning the current linguistic or phonetic goals and preferences (favorite songs, toys or other preferences) of the child was collected. This information was used to plan the content of each group's activities. There were maximum six children in each intervention session where parents were always present.

The musical activities in the intervention followed the principles given in the article by Torppa and Huotilainen (2019), based on the finding from previous studies on musical activities and interventions in children with HL. The sessions also draw from learner-centred and inclusive music education, which aims to construct a fruitful learning environment for every participant in the group (Huhtinen-Hildén and Pitt 2018; Huhtinen-Hildén et al. 2024). The music sessions were always led by a speech therapist and a music pedagogue and students from similar fields in the guidance of experienced professionals. The emphasis was on singing, and every session included also pitch exercises (e.g., scales and tone slides embedded in different themes). Gestures related to pitch movements were used since these have been shown to be effective in learning singing vocal accuracy (Liao 2008). Children were encouraged to use their voices in playful manners and this was celebrated in the group, but no individual feedback was given for the children to avoid negative feelings related to singing accuracy. Some of the songs were targeted to learn clothes, animals and other concepts or phonemes which were goals of individual children (goals were set based on the intervention of the parents). The song “Twinkle twinkle little star” and songs related to the themes the children wished for were included in the intervention. Importantly, the music pedagogue assured that the musical activities were motivating and supported the agency of the children. The pedagogically constructed learning environment was focused to generate 1) the will to participate in activities, 2) courage to explore voice in the playful activities 3) reason to repeat and “train the skills”. Thus, the purpose was joint playful activity were meaningful learning takes place (Huhtinen-Hildén and Pitt, 2018; Huhtinen-Hildén et al. 2024). Colorful music instruments such as kantele (Finnish national string instrument, Finnish zither”; the body of the instrument is made of wood; the steel strings are plucked with fingers), and pictures were used to motivate children, to visualize pitch movements and to help them to understand the meanings of lyrics of songs and structure the sessions.

A new software was developed for the purposes of the present study (by laboratory engineer Tommi Makkonen). Tablet computers were distributed to families to be used only during the intervention, to enable children and parents to listen to the songs (audio-only) used in the speech-music group sessions, and to listen to the Lindfors foundation's MUKULA materials (adapted into XXX by the first author and music therapist Seija Laakso from those of music therapists Christine Rocca and Catherine Bowker, Mary Hare School) including pitch exercises (one octave tone slides, scales, repeating intervals) embedded in music stories (story books related to these were given for the families). The songs entered to the software were recorded by the group leaders. The group leaders of two intervention groups used also youtube channel (videos) to spread out the songs used in the sessions. The aim was to always send the songs already before they were introduced in the music sessions to allow pre-learning, however, this was not always possible. The tablets also contained Outloud oy's Fasteroid application, which visualizes for the child the vocal pitch movements, as well as a recording option so that the children could record their own song on the tablet. Twice a week, the tablet sent parents a questionnaire that mapped the amount of use of the Fasteroid application and the amount of singing at home. Music instruments such as claves (percussion instrument, consisting of two sticks, which are played by striking them together) and 5-string kantele ("Finnish zither"; the body of the instrument is made of wood; the steel strings are plucked with fingers) were sent to families and used in the music sessions. Parents were provided specific, written instructions on how to listen to the songs and do pitch exercises using Fasteroid and songs, and how to answer the questions sent by the software. After the intervention period, the tablets and instruments were returned to researchers.

Based on the questionnaires sent by the tablet software for parents (number of parents who answered to the questions = 8), they sang with their child face to face (mean 2.06 times, 5.46 minutes per day); some of the children played the Fasteroid or other computer games training pitch perception and accuracy of sung pitch production (mean 0.68 times, 1.91 minutes per day across children). Only few of the parents made pitch exercises given for parents to be conducted with their child (mean 0.46 min per day).

References:

Huhtinen-Hildén, L., Kivijärvi, S., Elomaa-Krapu, M., Isola, A.-M. (2024). Creating affordances for families with young children: Experiences of music education in social work. *International*

Journal of Music Education, 0.

Huhtinen-Hildén, L., & Pitt, J. (2018). *Taking a learner-centred approach to music education. Pedagogical pathways*. Routledge.

Liao, M.-Y. (2008). The effects of gesture use on young children’s pitch accuracy for singing tonal patterns. *International Journal of Music Education, 26,* 197–211.

Torppa, R., & Huotilainen, M. (2019). Why and how music can be used to rehabilitate and develop speech and language skills in hearing-impaired children. *Hearing Research, 380,* 108–122.
