# Supplementary material for: Improved Singing Accuracy in Children With Bilateral Hearing Devices With More Musical Activities and Better Verbal Fluency
Source: Ear Hear. 2026 Feb 3;47(4):925–35. doi: 10.1097/AUD.0000000000001786 (PMC13252975; doi:10.1097/AUD.0000000000001786)
Supplement: Supplementary file 2 [file aud-47-0925-s002.pdf]

Appendix B. Questions, response options and scales used for informal musical activities.

To help the reader, the questions and response options have been translated from Finnish to English language for this Appendix. Please note that the wording below are not verbatim translations, but instead translations that give the reader the closest approximation (in grammatically correct English) to the Finnish question.

For all music activity questions, the logic of the survey was so that the second question on the frequency of participation was only asked if the parent answered 'yes' to the first question as to whether the child participated in that particular activity (yes/no). Response options for the second question were: 0 = less than once in a month; 1 = once in a month; 2 = 2-3 times per month; 3 = once in a week; 4 =2-3 times per week; 5 = 4-6 times per week; 6= daily. If the parent answered "daily", then they were required to answer to an open question "If daily, how many times per day."

In the final scoring of all questions on Informal musical activity, if the parent answered no to the first question (please see below), then a score of '0' was allocated for statistical analyses for this child, and the answers to questions "daily" were scored as 6 = 1-3 times per day; 7 = 4-7 times pr day; 8 = 8 or more times per day). This led to a 9-point Likert scale (0 = not at all/less than monthly; 1 = once in a month; 2= 2-3 times per month; 3 = once per week; 4 = 2-3 times per week; 5 = 4-6 times per week; 6 = 1-3 times per day; 7 = 4-7 times pr day; 8 = 8 or more times per day).

Informal musical activities

Questions included in statistical analyses:

1. Listening to music (audio only):
A) Has your child ever listened to music at home or elsewhere from recordings (without visual support, e.g. in the car, when going to bed, while eating, while playing, during naps, etc.)?
B) How often has your child listened to music?
C) If daily, how many times per day?

2. Social musical activities:

A) Has your child ever participated in social (self-initiated, not directed) musical activities (e.g., imitation, making music in role-plays with friends or siblings)?

B) How often has your child participated in social music activities?

C) If daily, how many times per day?

3. Musical videos:

A) Has your child ever watched music programs or music videos (e.g., music episodes of children's programs, YouTube videos)?

B) How often has your child watched music programs or videos?

C) If daily, how many times per day?

4. Family music activities:

A) Has your family ever made music together (for example, sing or play)?

B) Has your child participated in making music together?

C) How often have you made music together?

D) If daily, how many times per day?

5. Independent music exploration using instruments:

A) Has your child ever explored music independently (e.g., played self-made instruments or bowls/pots/spoons)?

B) How often has your child made independent musical explorations?

C) If daily, how many times per day?

6. Creating/making up songs or music performances for play or fun:

A) Has your child ever sung songs (learned or self-invented) or made musical performances during playtime?

B) How often has your child made these musical performances during playtime?

C) If daily, how many times per day?

7. Dancing informally:

A) Has your child ever danced to the music on his own initiative?

B) How often your child has danced to the music on his own initiative?

C) If daily, how many times per day?

8. Singing by the child

A) Does the child sing at home in general (alone, with siblings or friends or parents etc., altogether)?

B) How often does the child sing at home in general (alone, with siblings or friends or parents etc., altogether)?

C) If daily, how many times per day?

Questions not included in statistical analyses:

The answers to the question Music online was not included due to missing data (more than half of the parents either did not answer these questions or reported that their children did not play any music games). The answers to the questions on parental singing were not asked since we did not measure the singing pitch accuracy of the parents.

9. Music online:

A) Has your child ever used online music programs, apps or games (e. g., Fasteroid, Pitch painter, apps for music instrument playing or singing, apps related to perception of pitch or music,)?

B) How often has your child used the aforementioned programs, apps or games?

C) If daily, how many times per day?

10. Singing with parents before the measurement

A) Did you (child's parents) sing face to face with the child during the previous year before T1/between the measurements?

B) How often have the child's parents sung face to face with their child during the previous year/between the measurements?

C) If daily, how many times per day?

11. Singing with parents during the child's first year of hearing

A) Did you (child's parents) sing face to face with the child during the child's first year after activation of the HA/CI?

B) How often have the child's parents sung face to face with their child during the child's first year after activation of the HA/CI?

C) If daily, how many times per day?
