# Supplementary material for: Improved Singing Accuracy in Children With Bilateral Hearing Devices With More Musical Activities and Better Verbal Fluency
Source: Ear Hear. 2026 Feb 3;47(4):925–35. doi: 10.1097/AUD.0000000000001786 (PMC13252975; doi:10.1097/AUD.0000000000001786)
Supplement: Supplementary file 3 [file aud-47-0925-s003.pdf]

Appendix C. Supplementary Table 1

Descriptives for the semantic verbal fluency performance (number of correct words for the animal category) of children with hearing loss (HL group)

	T1 (n = 16)<br>M (SD)	T2 (n = 16)<br>M (SD)	T3 (n = 15)<br>M (SD)	Mean of T1, T2 and T3<br>M (SD) (N = 17)
Semantic verbal fluency performance	6.44 (4.79)	9.44 (5.56)	10.07 (6.42)	8.46 (5.66)

T1, T2, T3 = first, second, third time points of measurements; n = number of children performing the task.
